# Three-year continuous maize cropping improves saline-alkali soil microenvironment and crop productivity

**DOI:** 10.3389/fmicb.2025.1731672

**Published:** 2026-04-16

**Authors:** Lei Ling, Rixin Wang, Mingyi Wang, Naiyu Chen, Guohui Xu, Yirui Wang, Guoling Ren

**Affiliations:** 1 Heilongjiang Provincial Key Laboratory of Oilfield Applied Chemistry and Technology, Daqing, China; 2 College of Bioengineering, Daqing Normal University, Daqing, China; 3 College of Life Health Science and Technology, Dalian University, Dalian, China

**Keywords:** crop continuously, maize, microbial diversity, soda saline-alkali land, soil improvement

## Abstract

**Introduction:**

The efficient utilization of soda saline-alkali land is of great strategic significance for ensuring China's food security and improving the ecological environment. Cultivating salt-alkali tolerant plants can ameliorate the properties of saline-alkali soil, with rhizosphere microorsganisms playing a crucial role in this process.

**Methods:**

We conducted a 3-year field experiment in Lamadian, Daqing, China, using high-throughput sequencing to analyze the maize rhizosphere microbial community diversity and its relationships with soil properties, enzyme activities, and plant yield.

**Results:**

The results showed that pH and total salt (TS) content decreased annually, while the contents of available potassium (AK), available nitrogen, and available phosphorus (AP) and soil polyphenol oxidase (S-PPO), soil urease (S-UE), soil catalase, soil alkaline phosphatase, soil deoxyribonuclease, and soil sucrase (S-SC) enzyme activities increased yearly. The maize yield increased by 8.38% and 2.42% annually. Actinobacteriota, Proteobacteria, Acidobacteriota, and Chloroflexi were the dominant bacterial phyla in the maize rhizosphere soil. Ascomycota, Basidiomycota, and Mortierellomycota were the dominant fungal phyla. Correlation analysis indicated that *Blastococcus, Bacillus*, and *Nocardioides* were the key bacteria influencing AK, TS, S-UE, and S-PPO activity. In the fungal community, *Tausonia, Mortierella*, and *Gibellulopsis* were the key factors affecting AP, AK, and TS content, as well as S-SC, S-UE, and S-PPO activities.

**Conclusion:**

Three years of maize cultivation effectively improved the physicochemical properties and enzyme activities of saline-alkali soil and drove the restructuring of the rhizosphere microbial community. Notably, the fungal community structure tended to stabilize after 1 year, whereas bacterial diversity increased annually, revealing their distinct roles in ecological restoration. These results provide a theoretical basis and practical guidance for leveraging crop–microbe interactions to ameliorate saline-alkali land.

## Introduction

Soil salinization is a serious problem in global agriculture as it threatens the sustainability of crop production (Ricroch and Hénard-Damave, 2015; Bai et al., 2026). Currently, the global area of salinized soil is up to 1.13 × 109 hm^2^ (Guo et al., 2022; Ruiz-Lozano et al., 2012). The Songnen Plain is one of the regions in China that is most severely affected by salinization, which greatly affects agriculture (Yin et al., 2017). The soils in this region have a high pH, high exchangeable sodium content, soil compaction, and poor permeability. These conditions lead to growth inhibition and yield reduction in many crops (Song et al., 2025). Planting suitable crops can absorb, transform, and transport soil salts, thereby improving soil quality (Wang et al., 2018) and maintaining a stable and lasting desalination effect (Ma et al., 2014). Therefore, phytoremediation is currently one of the most cost-effective approaches for ameliorating saline-alkaline lands.

Maize (*Zea mays* L), with its strong adaptability and high annual yield, is cultivated worldwide and is a key crop for ensuring food security in China (Chen et al., 2025). Currently, the global maize planting area exceeds 1.41 × 108 hm^2^ (Shen et al., 2018). Maize rhizosphere microorganisms exhibit strong salinity-alkalinity tolerance. At the same time, they dissolve the insoluble phosphates in the soil to provide the nutrients needed for the growth of maize (Luo et al., 2024; Shang et al., 2025). Furthermore, the endophytic bacterium *Pseudomonas mendocina* M1 in maize roots reduces reactive oxygen species (ROS) content through an antioxidant system, alleviating saline-alkali stress (Li et al., 2024). Thus, the interaction between maize and rhizosphere microorganisms significantly enhances saline-alkali stress resistance, thereby promoting sustainable maize production in saline-alkali soils.

The rhizosphere is a critical site for plant-soil- microorganism interactions. In particular, rhizosphere microorganisms in the soil system drive key processes, such as the decomposition of soil organic matter and the transformation and cycling of soil nutrients (Gupta et al., 2022; Liu et al., 2021). Under saline-alkali stress, crops such as wheat (Sui et al., 2024) and rice (Lei et al., 2025) enrich beneficial rhizosphere microorganisms, reduce soil salinity, and enhance crop stress resistance. Soil enzymes mainly originate from microorganisms and are involved in the material metabolism and energy conversion in the soil, being closely related to soil fertility levels and microbial activity (Yang et al., 2023). Meanwhile, the dynamic changes in soil microbial communities can also significantly affect the activity of soil enzymes (Liu et al., 2011). Therefore, in the soil-plant system, rhizosphere microorganisms and soil enzymes collaboratively respond to saline-alkali stress, and their interactions constitute a key mechanism in resisting saline-alkali stress (Wang et al., 2021; Guo et al., 2021).

Current research has largely focused on the salt-alkali tolerance mechanisms of maize rhizosphere microorganisms and the isolation of relevant strains (Cui et al., 2023; Wallace et al., 2025; He et al., 2024), whereas the relationships among long-term continuous cropping, the dynamics of the maize rhizosphere microbial community, and saline-alkali soil remediation remain underexplored. Therefore, this study hypothesizes that three consecutive years of cultivation can ameliorate saline-alkali soil, as evaluated through soil physicochemical properties, enzyme activities, and maize yield. It aims to provide a theoretical basis for using rhizosphere microorganisms to enhance sustainable agricultural development on saline-alkali land.

## Methods

### Experimental materials and design

The selected test material was Zhengdan 958. The field experiment was conducted in Lamadian, Daqing City, Heilongjiang Province (46°42I03″N, 124°47I04″E). A randomized block design was employed with three replicates per treatment annually, resulting in a total of twelve experimental plots. Each plot area was 50 m^2^. The planting density was 60,000 plants/ha, with row spacing of 60 cm and plant spacing of 25 cm. Sowing was conducted in mid-May and harvesting in mid-September each year. Other field management practices followed conventional farming protocols.

### Yield measurement and sample collection

At maize maturity, 20 plants were randomly selected per plot, with three replications per year, resulting in a total of 60 plants collected annually. Ear number, kernels per ear, and 1,000-kernel weight were measured. Maize yield was calculated based on the three-year dataset. Control (CK) soil was collected from the same site prior to maize planting. After removing the 0–5 cm topsoil, maize root systems were excavated. A “soil shaking-off method” was applied to remove loosely adhered soil, followed by gentle brushing of the soil still attached to the roots. The collected soil was placed into sterile zip-lock bags and transported to the laboratory (Yao et al., 2025). One portion of the soil samples was air-dried, ground, and passed through a 1 mm sieve for determination of soil physicochemical properties and enzyme activities. Another portion was stored at −80 °C in an ultra-low temperature freezer for microbial diversity analysis.

### Soil physicochemical properties and enzyme activity analyses

Soil pH was measured using the electrode method. Soil organic matter was determined by the potassium dichromate volumetric method. Soil salt content was analyzed via deionized water extraction and mass quantification. Total nitrogen was assessed by the Kjeldahl method. Available phosphorus was measured using Mo-Sb anti-colorimetry. Available nitrogen was determined by alkaline hydrolysis diffusion. Available potassium was analyzed with flame photometry.

Soil enzyme activities were determined according to the methods described by Guan Songyin (Guan, 1986). Catalase, sucrose, urease, dehydrogenase, polyphenol oxidase, and alkaline phosphatase activities were measured using potassium permanganate titration, 3,5-dinitrosalicylic acid colorimetry, indophenol blue colorimetry, TTC colorimetry, o-phenylenediamine colorimetry, and p-nitrophenol colorimetry, respectively.

### Soil microbial high-throughput sequencing

Total DNA of soil bacteria and fungi was extracted using a Soil DNA Kit (Omega Bio-tek Inc., Doraville, GA, USA). The quality of the extracted microbial DNA was detected by 1% agarose gel electrophoresis. The DNA was then sent to Shanghai Majorbio Bio-pharm Technology Co., Ltd. for Mi Seq high-throughput sequencing. The bacterial 16S rDNA a mplification primers were 338F-806R (F: 5′-ACTCCTACG GGAGGCAGCA-3′, R: 5′-GGACTACHVGGGTWTCTAA T-3′). The fungal amplification primers were ITS1F-ITS2R (F: 5′-CTTGGTCATTTAGAGGAAGTAA-3′, R: 5′-GCTG CGTTCTTCATCGATGC-3′).

### Data analysis

Valid sequences were clustered into ASVs (≥97% similarity) using QIIME. Taxonomic annotation was performed with the Mothur method against the SILVA SSUrRNA database (threshold 0.8–1.0). Microbial community composition was statistically analyzed at phylum, class, order, family, and genus levels. Taxonomic bar plots and heatmaps were generated based on taxonomic analysis. The analysis of alpha diversity employed the nonparametric Kruskal-Wallis rank-sum test to evaluate differences between groups. For beta diversity, the ASV abundance table was normalized using Z-score transformation. PCA was then performed based on the Euclidean distance matrix, and permutational multivariate analysis of variance (Adonis) was used to test for significant differences in overall community structure.Differentially abundant taxa were screened with the Kruskal–Wallis test. Pairwise group comparisons were performed using the Wilcoxon rank-sum test. The effect size of each discriminant feature was estimated by linear discriminant analysis (LDA), with an LDA score > 4.0 considered significant (Han et al., 2024). Pearson correlation analysis was performed using SPSS 22.0. Differentially expressed enzyme activities were identified using multi- group comparison bar charts on the Majorbio Cloud Platform. Dynamic correlation heatmaps analyzed relationships among soil microbial diversity, physicochemical properties, and enzyme activities (Mu et al., 2024). Raw data were processed and visualized with Excel 2010.

## Results

### Changes in maize yield and enzyme activities in rhizosphere soil under saline-alkali conditions

As shown in Table 1, the soil pH and TS content in the maize rhizosphere decreased with increased planting years. Over 3 years of monoculture, the yearly pH values were 8.4, 8.03, and 7.78, with that in the third year being significantly lower than the first two. TS content decreased significantly by 20.24% and 19.60% in the second and third years, respectively. TN content decreased by 14.10% in the second year and increased by 44.34% in the third year. Available potassium (AK) content increased significantly by 23.90% and 19.75% in the second and third years, respectively. AP and AN content also increased annually but without significant differences. SOM content showed no significant changes.

**Table 1:** Physicochemical properties of the rhizosphere soil.

Treament	pH	SOM (mg ·g^−1^)	TS content (g·kg^−1^ )	TN content (g·kg^−1^ )	AP content (mg·kg^−1^ )	AN content (mg·kg^−1^ )	AK content (mg·kg^−1^ )
1-Year	8.40 ± 0.67	21.26 ± 4.06	179.33 ± 13.55	1402.00 ± 409.71	102.07 ± 1.75	53.11 ± 10.69	28.12 ± 3.51
2-Year	8.03 ± 0.16	21.85 ± 3.25	143.03 ± 16.96	1204.32 ± 79.45	103.30 ± 3.26	67.34 ± 13.72	34.84 ± 3.70
3-Year	7.78 ± 0.39	21.26 ± 4.06	115.00 ± 20.26	1738.35 ± 139.96	111.87 ± 2.36	67.76 ± 4.21	41.72 ± 2.82

SOM, soil organic matter; TS, total salt; TN, total nitrogen; AP, available phosphorus; AN, available nitrogen; AK, available potassium.

**Figure 1:**
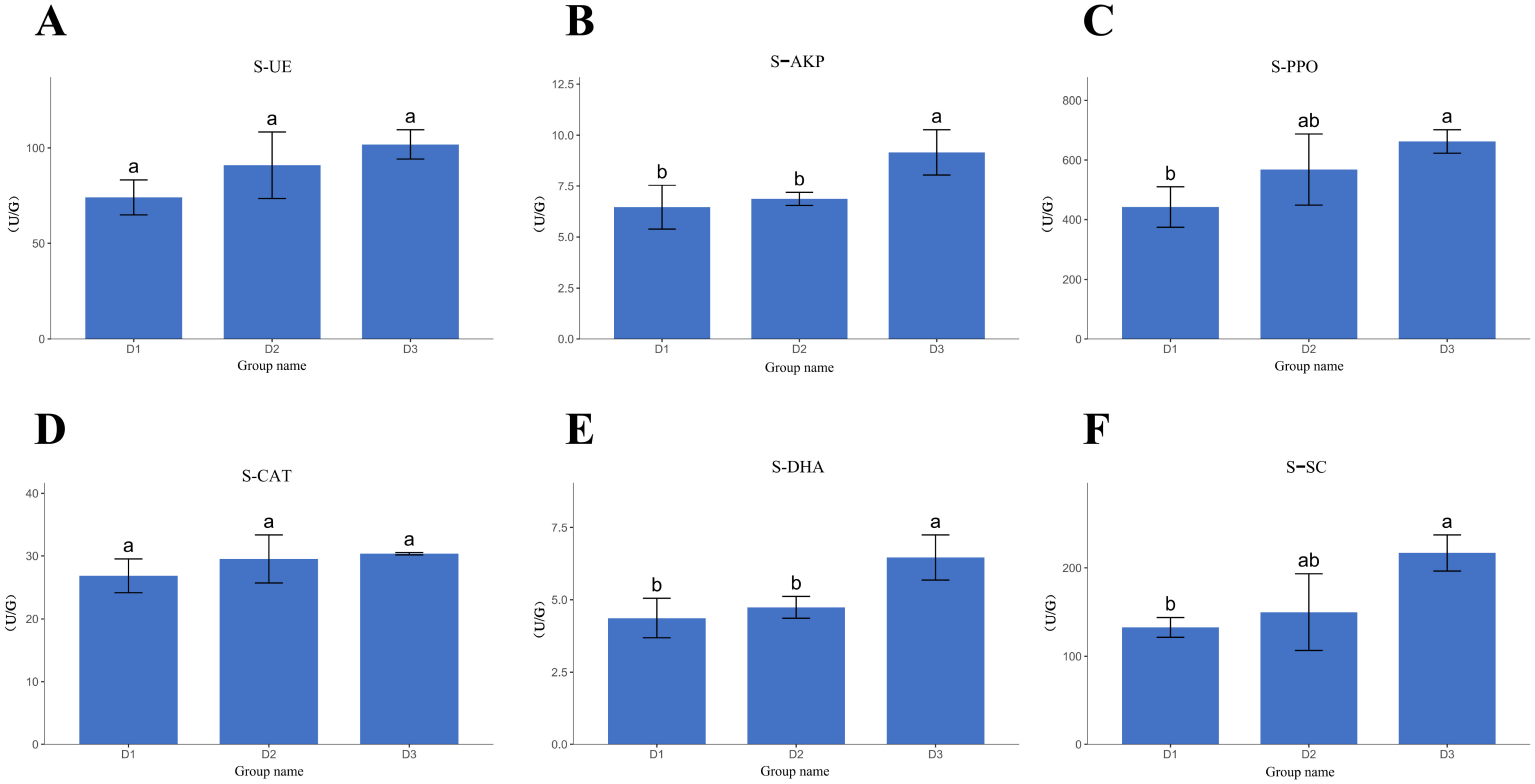
Differences in enzyme activity in maize rhizosphere soil. **(A)** S-CAT, soil catalase; **(B)** S-PPO, soil polyphenol oxidase; **(C)** S-SC, soil sucrase; **(D)** S-DNA, soil dehydrogenase; **(E)** S-UE, soil urease; **(F)** S-AKP, soil alkaline phosphatase. Different lowercase letters indicate significant differences at *p* < 0.05.

Analysis of maize rhizosphere soil enzyme activity (Figure 1) revealed that the activities of all six enzymes increased annually. S-PPO and S-UE increased annually, with S-PPO increasing by 28.50% and 16.64% (*p* < 0.05), and S-UE by 15.70% and 12.03% (*p* < 0.05) in the second year, respectively. The S-DHA, S-AKP, and S-SC levels increased significantly by 47.83%, 41.80%, and 63.4% (*p* < 0.05) in the third year, respectively. S-CAT showed no significant differences. The 3-year consecutive maize planting treatment effectively enhanced soil enzyme activity.

### Effects on maize yield and yield components in saline-alkali soil

**Figure 2:**
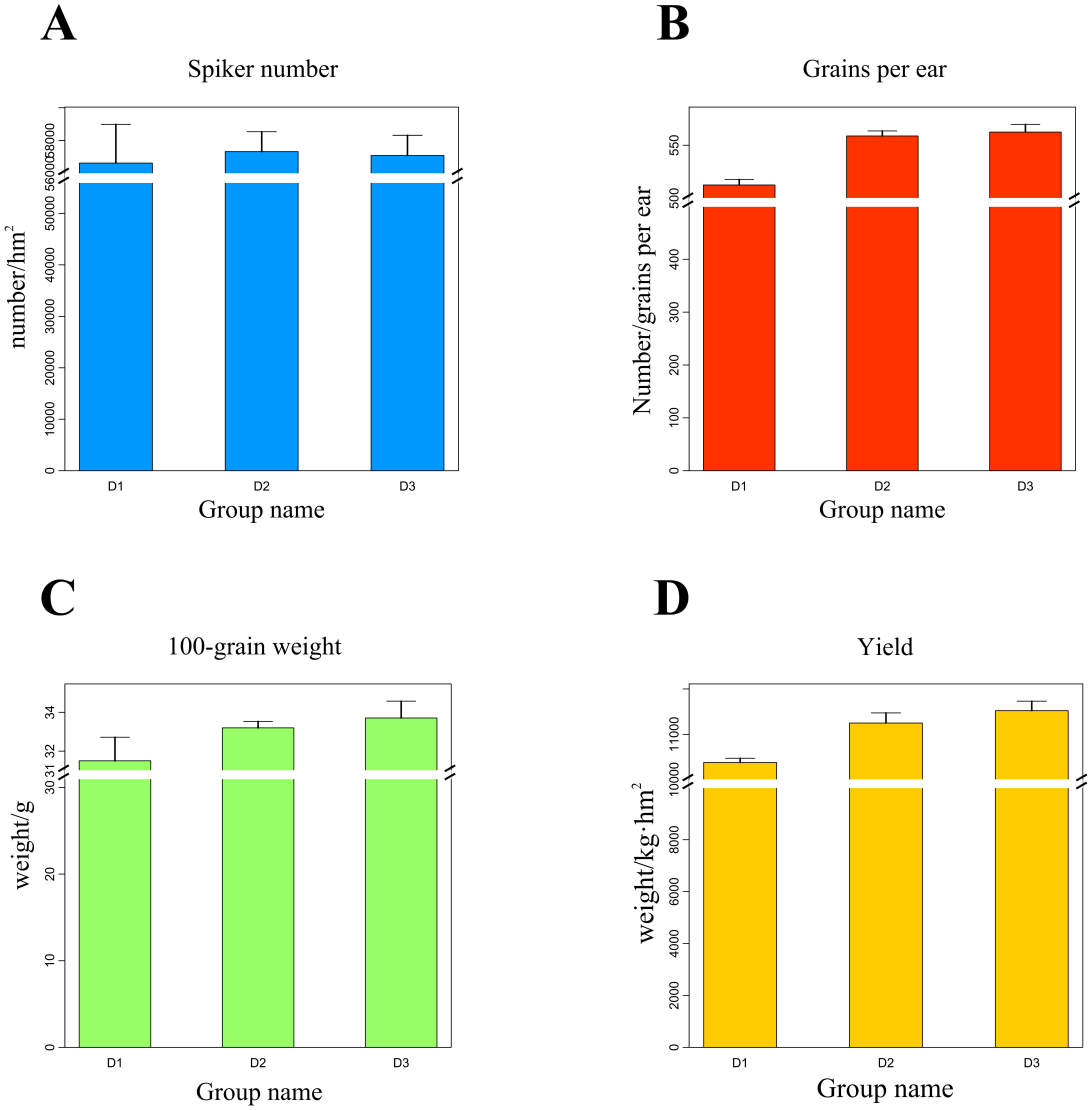
Effect of 3-year monoculture treatment on maize yield. **(A)** Number of maize ears, **(B)** number of grains per ear, **(C)** 100-grain maize weight, **(D)** maize yield.

As shown in Figure 2, the yield components of maize showed significant changes among planting years. The number of ears per plant exhibited an initial increase followed by a decrease. The number of ears increased by 1.22% and decreased by 0.40% annually, showing an initial increase followed by a decline. Kernel number per ear increased in the second and third year by 8.99% and 0.66%, respectively. The hundred-kernel weight increased by 5.48% and 1.42%, respectively. Additionally, the increase in the number of grains per ear and the weight of 100 grains was not significant in the following 2 years. The maize yield increased significantly by 8.38% and 2.42% in the second and third year, respectively.

### Analysis of the α and β diversity of maize rhizosphere soil microorganisms

**Figure 3:**
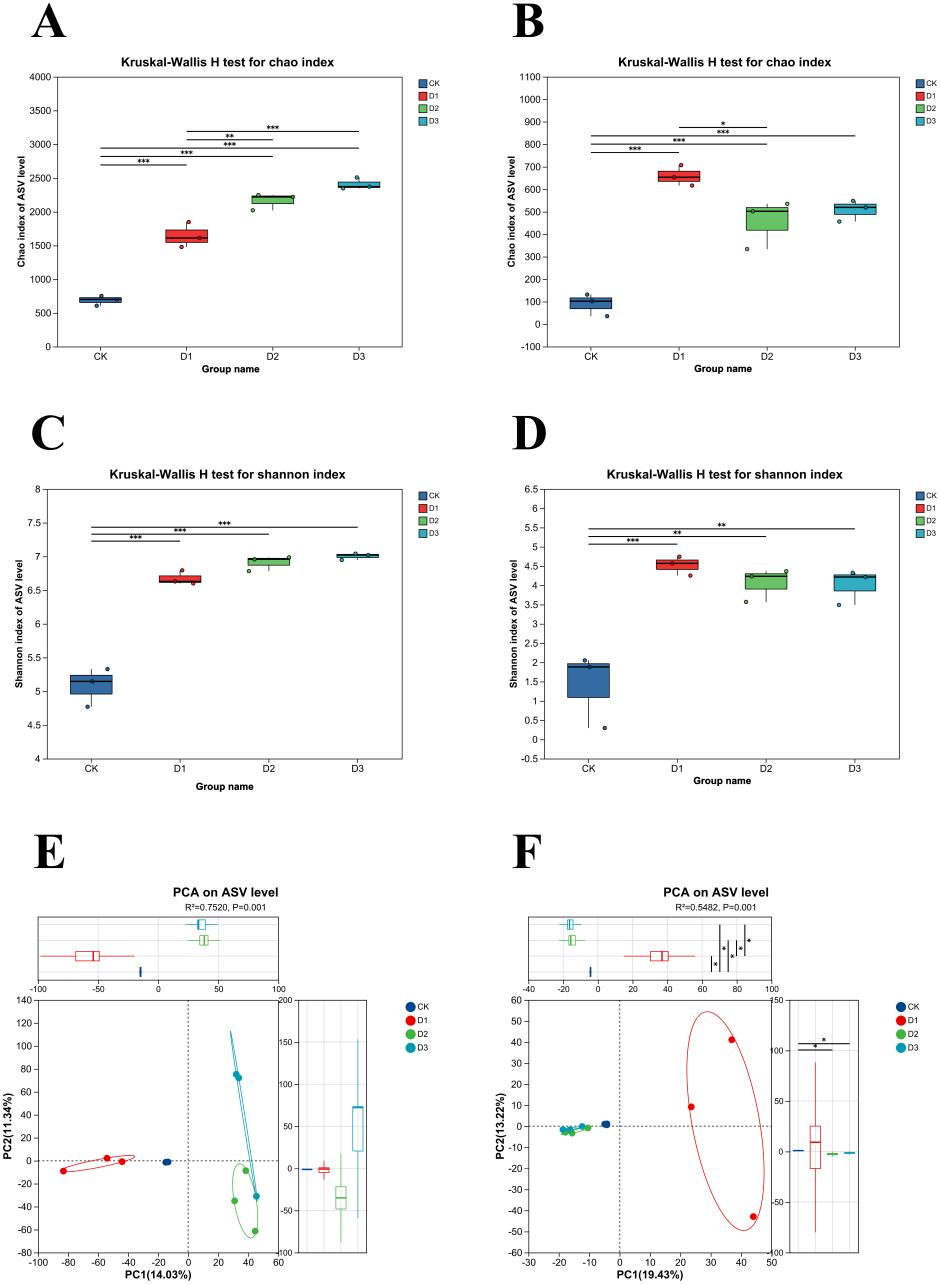
Analysis of the alpha and beta diversities of microbial communities in the rhizosphere of maize. Chao index of bacterial **(A)** and fungal **(B)** diversity in rhizosphere soil, Shannon index of bacterial **(C)** and fungal **(D)** diversity in rhizosphere soil, PCA analysis of bacterial **(E)** and fungal **(F)** communities in rhizosphere soil.

Significant differences were observed in the Chao and Shannon indices of both bacterial and fungal communities across three consecutive years of cultivation. As shown in Figures 3A, C, the bacterial Chao and Shannon indices increased significantly each year, indicating a rising trend in both bacterial diversity and richness. In contrast, the fungal Chao and Shannon indices peaked in the first year and then stabilized, with no significant differences between the latter 2 years (Figures 3B, D). This suggests that the fungal community rapidly adapted to form a stable structure, whereas the bacterial response was slower and persisted longer.

Principal component analysis of the maize rhizosphere microbial communities was performed at the ASV-level. Figure 3E shows that the contribution rates of the principal components PC1 and PC2 for the bacterial samples were 14.03% and 11.34%, respectively, with a total contribution rate of 25.37%. The CK and first-year samples are distributed on the negative axis of PCA1, whereas samples from the latter 2 years are distributed on the positive axis, with partial overlap. Figure 3F shows that the contribution rates of the principal components PC1 and PC2 to the fungal samples were 19.43% and 13.22%, respectively, with a total contribution rate of 32.65%.Only the first-year samples were distributed in the positive direction of the PC1 axis, while the samples from the second and third years were located in the negative direction, exhibited substantial mutual overlap, and were clearly separated from the first-year samples. This indicates that the differences in fungal community structure progressively diminished. A similar trend was observed for the bacterial community structure. Together, these findings suggest that the maize rhizosphere microbial community underwent rapid restructuring during the initial remediation phase, followed by stabilization.

### Analysis of soil microbial community structure

**Figure 4:**
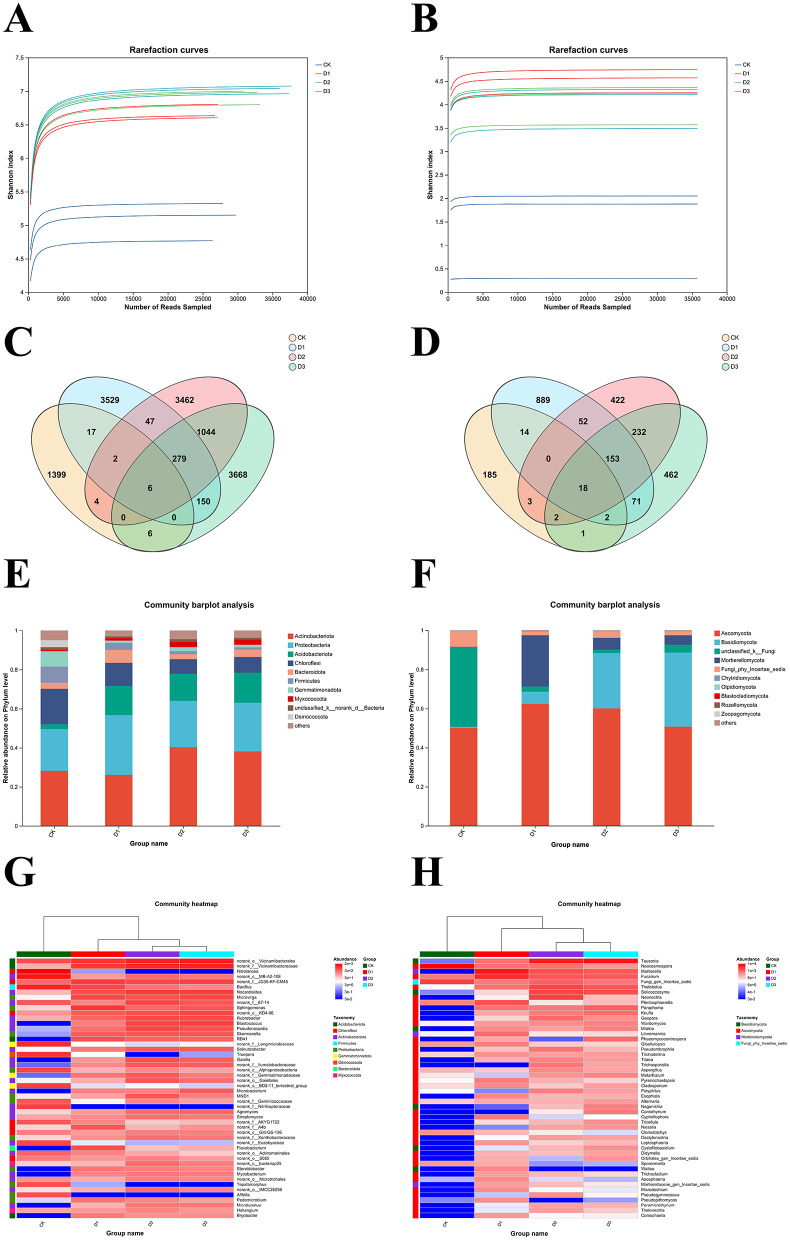
Relative abundance distribution of maize rhizosphere soil microbial communities. Dilution curve of rhizosphere soil bacterial **(A)** and fungal community **(B)**. Venn diagram of ASV-level bacteria **(C)** and fungi **(D)** in the rhizosphere soil, soil bacterial communities of maize at the phylum **(E)** and genus **(G)** level, and soil fungal communities of maize at the phylum **(F)** and genus **(H)** level.

With the increase in the amount of sequencing data, the dilution curve of soil samples from the maize rhizosphere gradually flattened, which indicated that the sequencing depth had reached a certain level, the sampling was reasonable, and the microbial structure of soil samples from the maize rhizosphere was credible and may reflect the microbial community structure of all soil samples (Figures 4A, B). Venn analysis was performed on the maize rhizosphere soil microorganisms. Figure 4C shows that the numbers of bacterial ASVs in the CK and years 1, 2, and 3 were 434, 4,030, 4,842, and 5,153, respectively. There were six shared ASVs and 1,399, 3,529, 3,462, and 3,668 unique ASVs, respectively. The bacterial community abundance showed a yearly increase during the 3-year treatment. Figure 4D shows that the numbers of fungal ASVs for the CK and Years 1, 2, and 3 were 225, 1, 199, 882, and 941, respectively. There were 18 shared ASVs and 185, 889, 422, and 462 unique ASVs. Fungal community abundance was the highest in the first year. Although the abundance in the latter 2 years was lower than that in the first year, it was significantly higher than that in the CK.

Figure 4E shows that the dominant bacterial phyla were Actinobacteria, Proteobacteria, Acidobacteria, and Chloroflexi, which collectively accounted for over 83% of the total abundance across the 3 years. The abundance of Actinobacteria decreased by 2.1% in the first year and then increased by 12.10% and 9.93% in the two subsequent years. The abundances of Proteobacteria and Acidobacteria increased annually by 9.24%, 2.38%, and 3.55% and those of Acidobacteria increased by 12.44%, 11.42%, and 12.98%. The abundance of Chloroflexi decreased by 6.29%, 10.12%, and 10.12%.

Figure 4F shows that the dominant fungal phyla were Ascomycota, Basidiomycota, and Mortierellomycota, which collectively accounted for over 93% of the total abundance of all three phyla increased: that of Ascomycota by 12.38%, 10.05%, and 0.2%; Basidiomycota by 5.57%, 27.74%, and 37.25%; and Mortierellomycota by 26.1%, 5.88%, and 4.7% in each of the 3 years, with the highest increase observed in the first year. Unclassified_k_Fungi accounted for 40.83% of the abundance in CK but decreased significantly by 38.18%, 39.1%, and 36.82% over the 3 years.

Hierarchical clustering analysis and heatmap visualization were performed for the soil microorganisms. For bacterial genera (Figure 4G), the abundances of *Blastococcus, Gaiella, Microbacterium, Mycobacterium, Steroidobacter, Bryobacter*, and RB41 increased over the three years, whereas the abundance of *Nitrolancea, norank_f__Nitriliruptoraceae, Tepidamorphus*, and *Afifella* decreased. Among the fungal genera (Figure 4H), *Tausonia* and *Mortierella* increased over the three years. The abundances of *Titaea, Coniothyrium*, and *Naganishia* increased in the latter two years, whereas those of *Pseudopithomyces* and *Waitea* increased only in the first year.

### Analysis of the changes in microbial composition in the maize rhizosphere

**Figure 5:**
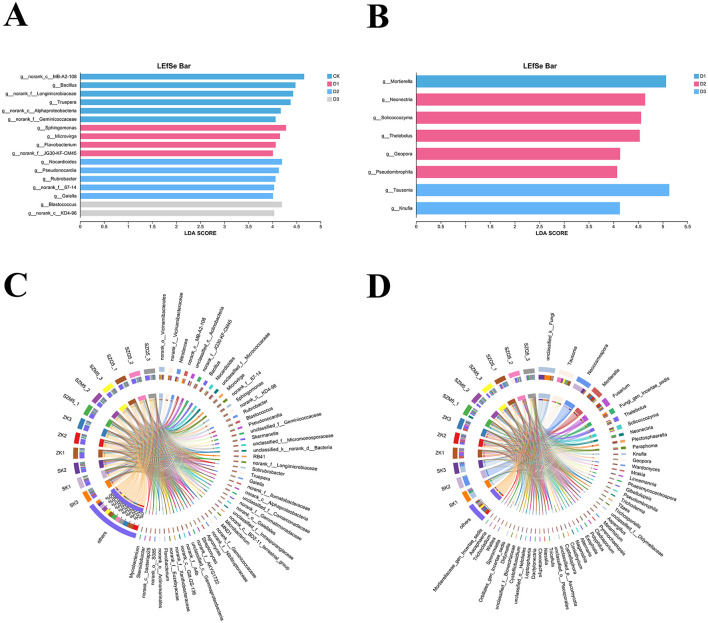
Differences in dominant bacteria and fungi groups at the genus level. LEfSe analysis of rhizosphere bacterial **(A)** and fungal **(B)** diversity, proportion, and distribution of dominant bacterial **(C)** and fungal **(D)** genera in maize rhizosphere soil.

LEfSe was used to identify significantly differentially expressed biomarkers at the genus level before and after maize cultivation (LDA threshold 4, *p* < 0.05). Figure 5A shows 17 differentially expressed bacterial taxa. Among them, CK had six biomarker microorganisms: *MB-A2-108, Bacillus, Longimicrobiaceae, Truepera, Alphaproteobacteria*, and *Geminicoccaceae*; Year 1 had four biomarkers: including *Sphingomonas, Microvirga, Flavobacterium*, and *JG30-KF-CM45*; Year 2 had five biomarkers: *Nocardioides, Pseudonocardia, Rubrobacter, g__norank_f__67-14*, and *Gaiella*; Year 3 had two biomarkers: *Blastococcus* and *g__norank_c__KD4-96*.

Figure 5B shows that eight differential fungal taxa were identified. Year 1 had only one biomarker, *Mortierella*; Year 2 had five biomarkers, including *Neonectria, Solicoccozyma, Thelebolus, Geopora*, and *Pseudombrophila*; and Year 3 had two biomarkers, including *Tausonia* and *Knufia*. No biomarker was detected in the CK.

At the genus level (Figures 5C, D), the dominant bacteriain the maize rhizosphere were *Vicinamibacterales, Vicinamibacteraceae*, and *Nitrolancea*, whereas the dominant fungi were *unclassified_k__Fungi, Tausonia, Neocosmospora*, and *Mortierella*.

### Relationship between soil microbial communities and physicochemical properties/enzyme activities

**Figure 6:**
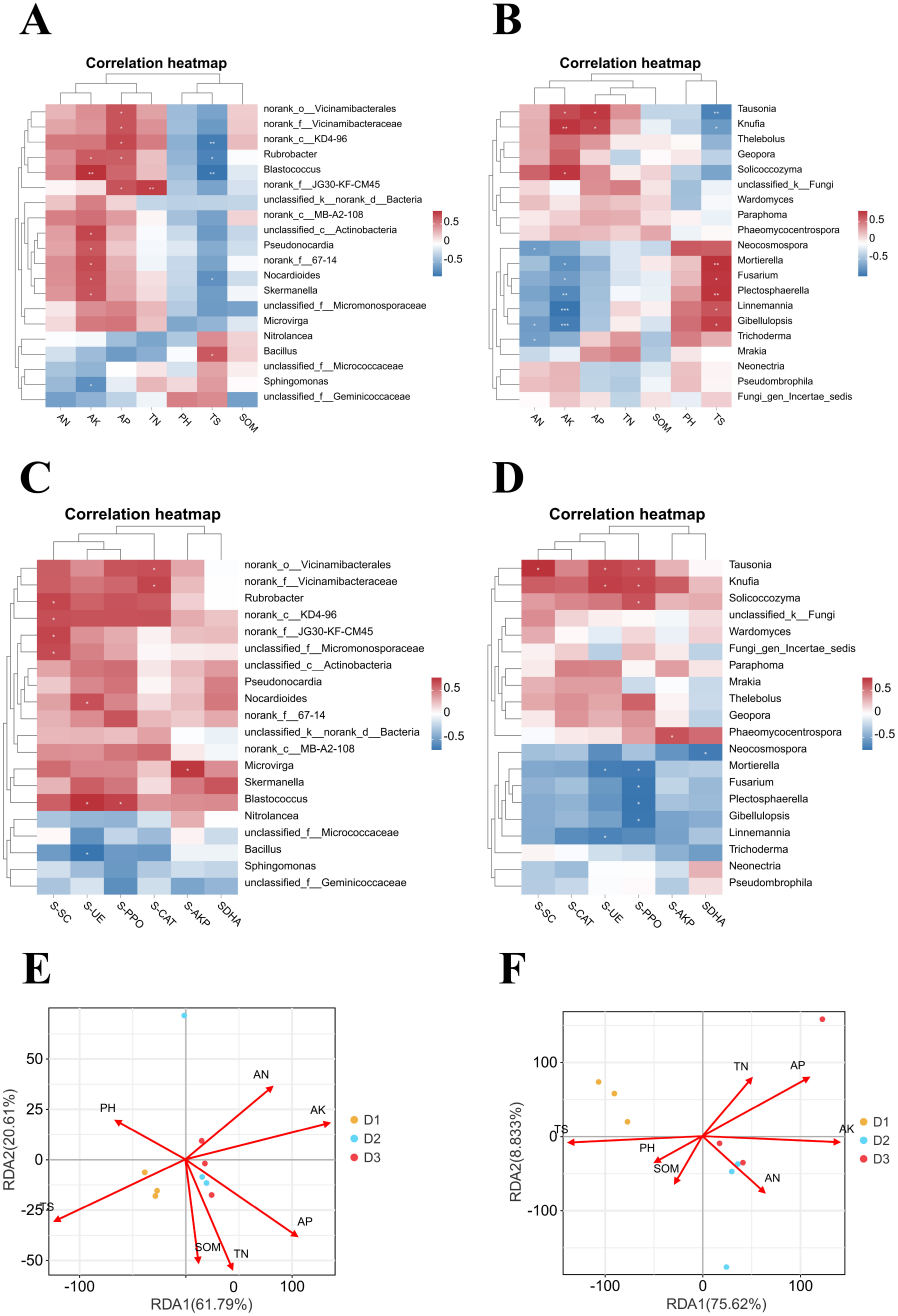
Correlation analysis of soil physical and chemical properties, enzyme activity, and rhizosphere microorganisms. Correlation analysis of the top 20 bacterial **(A)** and fungal **(B)** genera and soil physicochemical properties, correlation analysis of the top 20 bacterial **(C)** and fungal **(D)** genera and enzyme activity, redundancy analysis of bacterial **(E)** and fungal **(F)** communities and environmental factors at the genus level. White asterisks indicate statistical significance: *p < 0.05, **p < 0.01, ***p < 0.001. ns, not significant.

Correlation analysis between the top 20 genera and physicochemical properties revealed the following: for bacteria (Figure 6A), AK showed a highly significant positive correlation with *Blastococcus* (*p* < 0.01) and significant positive correlations with *Rubrobacter, unclassified_c_Actinobacteria, Pseudonocardia, norank_f_67-14, Nocardioides*, and *Skermanella* (*p* < 0.05), but a significant negative correlation with *Sphingomonas*. AP was significantly and positively correlated with *norank_o_Vicinamibacterales, norank_f_Vicinamibacteraceae, norank_c_KD4-96, Rubrobacter*, and norank_f_JG30-KF-CM45 (*p* < 0.05). TN exhibited a highly significant positive correlation with *norank_f_JG30-KF-CM45* (*p* < 0.01). TS showed a significant positive correlation with *Bacillus* (*p* < 0.05), highly significant negative correlations with *norank_c_KD4-96* and *Blastococcus*, and significant negative correlations with *Rubrobacter* and *Nocardioides* (*p* < 0.01). No significant correlations were observed between AN, pH, or SOM. For fungi (Figure 6B), AN showed a significant negative correlation with *Neocosmospora, Gibellulopsis*, and *Trichoderma* (*p* < 0.05). AK showed a highly significant positive correlation with *Knufia* (*p* < 0.01), significant positive correlations with *Tausonia* and *Solicoccozyma* (*p* < 0.05), highly significant negative correlations with *Linnemannia, Gibellulopsis*, and *Plectosphaerella* (*p* < 0.001), and significant negative correlations with *Mortierella* and *Fusarium* (*p* < 0.05). Notably, eight genera were significantly correlated with AK and exhibited opposite relationships with TS. AP showed a significantly positive correlation with *Tausonia* and *Knufia* (*p* < 0.05). No significant correlations were observed among TN, SOM, or pH.

For bacteria (Figure 6C), S-SC showed significant positive correlations with *Rubrobacter, norank_c_KD4-96, norank_f__JG30-KF-CM45*, and *unclassified_f_Micromonosporaceae* (*p* < 0.05). S-UE showed significant positive correlations with *Nocardioides* and *Blastococcus*, but a significant negative correlation with *Bacillus* (*p* < 0.05). S-PPO was significantly and positively correlated with *Blastococcus* (*p* < 0.05). S-CAT showed a significant positive correlation with *norank _o _ Vicinamibacterales* and *norank_f_ Vicinamibacteraceae* (*p* < 0.05). S-AKP expression was significantly and positively correlated with the presence of microviruses (*p* < 0.05). No significant correlation was observed for S- DHA. For fungi (Figure 6D), S-SC, S-UE, and S-PPO were significantly and positively correlated with *Tausonia* (*p* < 0.05). S-UE and S-PPO showed significant positive correlations with *Knufia* but significant negative correlations with *Mortierella* (*p* < 0.05). The S-UE was significantly negatively correlated with *Mortierella* and *Linnemannia* (*p* < 0.05). S-PPO was significantly negatively correlated with *Mortierella, Fusarium, Plectosphaerella*, and *Gibellulopsis* (*p* < 0.05). S-AKP was significantly positively correlated with *Phaeomycocentrospora* (*p* < 0.05). S-DHA levels were significantly negatively correlated with *Neonectria* (*p* < 0.05). No significant correlation was observed between microbial strains and S-CAT.

To further investigate the effects of soil environmental factors on the rhizosphere microbial community, an RDA was performed between soil environmental factors and bacterial and fungal genera was conducted (Figures 6E, F). The selected soil environmental factors explained 82.4% and 84.4% of the variation in the microbial communities, respectively. Based on the vector lengths in the ordination plot, AK and TS were the physicochemical factors with the strongest influence on the microbial community structures.

## Discussion

High total salt content and elevated pH are two key limiting factors in crop growth in saline-alkali soils (Tian et al., 2025). In this study, soil pH decreased by 4.40% and 3.11% annually with increasing maize planting years, whereas TS decreased by 20.24% and 19.60% annually, indicating that maize effectively reduced soil salinity. Sucrase promotes carbon cycling in the soil and catalyzes organic matter decomposition (Kotroczó et al., 2014), whereas urease facilitates the hydrolysis of nitrogen-containing organic compounds, increasing the soil nitrogen and available nitrogen content (Liang et al., 2003). The sucrase activity in the maize rhizosphere increased by 12.96% and 44.62% annually, whereas the urease activity increased by 22.7% and 12.0%, respectively. The TN content decreased by 14.10% in the second year but increased by 44.34% in the third year, whereas the AN content increased by 26.79% and 0.62% annually. Maize improves soil health by enhancing soil enzyme activity. The diversity and dynamic balance of plant rhizosphere microbiota are key factors in ensuring healthy plant development (Xue et al., 2011).

Bacterial alpha diversity indices increased annually, whereas the fungal Shannon index stabilized after a significant increase in the first year. In saline-alkali soil, specific environmental conditions differentially influence bacteria and fungi (He et al., 2025). In this study, the fungal community structure stabilized more rapidly, while the bacterial community continued to develop alongside gradual improvements in soil physicochemical properties. This suggests that fungi may possess stronger environmental adaptation potential, while bacterial communities are more sensitive to changes in soil conditions, consistent with findings from Zhao et al. in their study on *Suaeda* salsa (Zhao et al., 2024). Principal component analysis (PCA) of beta diversity further showed that with increasing cultivation years, bacterial and fungal community structures exhibited high overlap in the later 2 years. Long-term cultivation may drive their synergy, ultimately forming a more stable micro-ecosystem, which is consistent with the results reported by Wang (2022).

Actinobacteria, Proteobacteria, Acidobacteria, and Chloroflexi were the dominant bacterial phyla. Actinobacteria and Proteobacteria have been widely reported to be the dominant microbial groups in various saline-alkali soils (Sun Y. et al., 2022; Yang et al., 2016) and rhizosphere soils of multiple plants (Yang et al., 2016; Mei et al., 2025). In the present study, maize cultivation increased the abundance of Actinobacteria and Proteobacteria, with Actinobacteria being the dominant phylum, followed by Proteobacteria. Actinobacteria include species capable of producing enzymes and organic acids as well as forming spores in response to external stress (Tian and Zhang, 2017). The high relative abundance of Proteobacteria indicates its potential to play an important role in system nutrient cycling. Multiple studies have shown that Proteobacteria contain numerous species involved in organic matter decomposition and nitrogen and phosphorus transformation (Wang et al., 2020; Dai et al., 2018). The *RB41* Acidobacteria strain plays a crucial role in maintaining the ecological stability and metabolism of soil under nutrient-poor or stress conditions (Foesel et al., 2013). Its increase in abundance over the three planting years in this study suggests that maize alters rhizosphere microbial abundance, thereby enhancing soil nutrient contents. Ascomycota, Basidiomycota, and Mortierellomycota were the dominant fungal phyla. Ascomycota and Basidiomycota are the major decomposers in soil (Yelle et al., 2008). Most Ascomycota species are saprophytic, therefore they decompose refractory organic matter and contribute to nutrient cycling (Beimforde et al., 2014; Zhang et al., 2024). In this study, Ascomycota was the most abundant phylum, showing an initial increase, followed by a decrease over the 3 years. In previous studies (Zhang et al., 2022; Sun R. et al., 2022), the Basidiomycota phylum constituted a significant subdominant bacterial community within the ecosystem, with its abundance increasing significantly by 5.57%, 22.17%, and 9.61% annually, which may be related to the strong adaptability of maize in saline-alkaline environments.

Significant correlations were observed between maize rhizosphere microorganisms, soil physicochemical properties, and enzyme activity. In the bacterial community, *Blastococcus* showed a significant positive correlation with AK and a significant negative correlation with pH. Given its potential for nutrient transport, stress tolerance, and plant growth promotion (Sbissi et al., 2025), *Blastococcus* may alleviate saline-alkali stress through mechanisms such as the secretion of extracellular polymeric substances and production of organic acids. In contrast, the common salt-alkali tolerant genus *Bacillus* exhibited a significant positive correlation with total salt content (Zhang et al., 2025; Bharti et al., 2015). Its relative abundance decreased with declining salinity, likely reflecting a weakened competitive advantage following stress alleviation (De León-Lorenzana et al., 2018), coupled with niche restructuring and competitive exclusion within the rhizosphere community driven by the proliferation of other microbial groups. This drives a shift in the community assembly mechanism from stress-dominated to nutrient competition-oriented succession. Conversely, *Bacillus* becomes a dominant species aiding alfalfa in adapting to saline-alkali stress. This comparison indicates that maize recruits and shapes a distinct microbial community, reflecting the host plant's selective shaping of its rhizosphere microbiota. Additionally, *Blastococcus* significantly and positively correlated with S-UE and S-PPO, whereas *Bacillus* significantly and negatively correlated with S-UE. In the fungal community, *Tausonia* showed a significant positive correlation with AK and AP, and a highly significant negative correlation with TS. Organic acids secreted by microorganisms can release fixed phosphorus and potassium nutrients and lower soil pH (Gao et al., 2018). Therefore, Tausonia may alleviate saline-alkali stress by producing organic acids. *Mortierella* exhibited a significant negative correlation with AK and an extremely significant positive correlation with TS, which is consistent with the findings of Bai et al. (2024) and Liu et al. (2022). The salt-alkali-tolerant *Mortierella*, which was dominant in the early stages of improvement, experienced niche displacement and reduced abundance due to environmental improvement and increased competition. Meanwhile, the increase in AK content is a direct result of improved soil physicochemical properties, leading to their negative correlation. This discrepancy may arise from differences in rhizosphere-enriched microorganisms among different plant species, resulting in varied adaptive mechanisms to salt-alkali stress. As a recognized pathogenic fungus (Yang et al., 2020), *Gibellulopsis* showed an extremely significant negative correlation with AK, a significant negative correlation with AN, and a significant positive correlation with TS in this study. Its abundance decreased annually, possibly because the soil environment during the process of corn-improved saline-alkali land is unfavorable for *Gibellulopsis* survival, indicating alleviation of salt-alkali stress. Furthermore, *Tausonia* was significantly positively correlated with S-SC, S-UE, and S-PPO; *Mortierella* was significantly negatively correlated with S-UE and S-PPO; and *Gibellulopsis* was significantly negatively correlated with S-PPO.

## Conclusion

To investigate the bioremediation potential of maize on saline-alkali soil, a 3-year field experiment was conducted in Daqing's soda saline-alkali land. The results show soil pH decreased by 4.40% and 3.11%, and total salt content decreased by 20.24% and 19.60%, respectively. Furthermore, soil enzyme activities increased annually, and maize yield improved by 8.38% and 2.42%, respectively. The fungal community structure stabilized rapidly, whereas the bacterial community continued to develop slowly. Notably, common salt-alkali tolerant taxa, such as *Bacillus* and *Mortierella*, were gradually replaced as salinity decreased. This indicates that maize drives the rhizosphere microbial community to effectively alleviate saline-alkali stress. The proliferation of diverse microbial groups triggered niche restructuring and competitive exclusion within the rhizosphere community, which caused a shift from stress-dominated to nutrient competition-oriented succession.

## Data Availability

The data presented in this study have been deposited in the NCBI Sequence Read Archive (SRA) under accession number PRJNA1367765.
